# Construction of a pIX-modified Adenovirus Vector Able to Effectively Bind to Nanoantibodies for Targeting

**Published:** 2014

**Authors:** M. N. Garas, S. V. Tillib, O. V. Zubkova, V. N. Rogozhin, T. I. Ivanova, L. A. Vasilev, D. Yu. Logunov, M. M. Shmarov, I. L. Tutykhina, I. B. Esmagambetov, I. Yu. Gribova, A. S. Bandelyuk, B. S. Naroditsky, A. L. Gintsburg

**Affiliations:** N.F. Gamaleya Research Institute of Epidemiology and Microbiology, Ministry of Health of the Russian Federation; Gamaleya Str., 18, Moscow, Russia, 123098; Institute of Gene Biology, Russian Academy of Sciences; Vavilova Str., 34/5, Moscow, Russia, 119334; K.I. Skryabin Moscow State Academy of Veterinary Medicine and Biotehnology, Akademik Skraybin Str., 23, Moscow, Russia, 109472

**Keywords:** adenoviral vector, pIX, leucine zipper, nanobody, CEA

## Abstract

Current targeting strategies for genetic vectors imply the creation of a specific vector for every targeted receptor, which is time-consuming and expensive. Therefore, the development of a universal vector system whose surface can specifically bind molecules to provide efficient targeting is of particular interest. In this study, we propose a new approach in creating targeted vectors based on the genome of human adenovirus serotype 5 carrying the modified gene of the capsid protein pIX (Ad5-EGFP-pIX-ER): recombinant pseudoadenoviral nanoparticles (RPANs). The surfaces of such RPANs are able to bind properly modified chimeric nanoantibodies that specifically recognize a particular target antigen (carcinoembryonic antigen (CEA)) with high affinity. The efficient binding of nanoantibodies (aCEA-RE) to the RPAN capsid surfaces has been demonstrated by ELISA. The ability of the constructed vector to deliver target genes has been confirmed by experiments with the tumor cell lines A549 and Lim1215 expressing CEA. It has been shown that Ad5-EGFP-pIX-ER carrying aCEA-RE on its surface penetrates into the tumor cell lines A549 and Lim1215 via the CAR-independent pathway three times more efficiently than unmodified RPAN and Ad5-EGFP-pIX-ER without nanoantibodies on the capsid surface. Thus, RPAN Ad5-EGFP-pIX-ER is a universal platform that may be useful for targeted gene delivery in specific cells due to “nanoantibody–modified RPAN” binding.

## INTRODUCTION

Recombinant pseudoadenoviral nanoparticles (RPANs), which are derived from the human adenovirus serotype 5 (Ad5) genome with deletion of the region responsible for replication, are considered to be among the most promising tools for targeted gene delivery into mammalian cells. RPANs are extensively used in recombinant vaccines and gene therapy [1, 2]. The fact that RPAN is safe has been confirmed in a number of clinical trials of Ad5-based vaccines and gene therapy products. Since 2008, a quarter of gene therapy clinical trials have utilized the Ad-based RPANs [3]. Furthermore, two Ad5-derived gene therapy products have already been approved in China. There are a number of advantages contributing to the popularity of Ad5-based RN APs: Ad5-based vectors transduce both dividing and non-dividing cells; adenovirus DNA does not integrate into the host genome but remains extrachromosomal; RPANs can be produced at titer of more than 1010 pfu/ml, which allows one to use them as live recombinant vaccines; RPANs provide a high expression level of the transferred gene in the targeted cells.

However, some limitations in the use of Ad5-based RPANs exist. For instance, efficiency in the transduction of some mammalian cell types, particularly human tumor cells, can be low. This is due to the fact that the primary receptor for Ad5---coxsackievirus and adenovirus receptor (CAR)---is not expressed in all cell types [4-6]. To provide targeted gene transfer into CAR-deficient and CAR-negative cells, tropism modification strategies that alter the components of the A5-capsid (namely, fiber, hexon, pIX, pIIIa proteins) have been developed. Nowadays, these strategies enable Ad5-based RPAN delivery in various cell types, in particular targeting cervical cancer, glioma, renal cell carcinoma, ovarian cancer, as well as vascular smooth muscle cells [7-12].

Lately, the minor capsid protein IX (pIX) has received considerable attention as a site for protein ligand integration into adenovirus capsid. There are several advantages to pIX modification: the possibility to integrate relatively large peptide fragments to the Cterminus of pIX; the high structural compatibility of ligands with pIX; and a wide range of applications for Ad-based vectors with modified pIX [13].

It has recently been shown that integration of the RGD-motif (arginine-glycine-aspartic acid) into the pIX structure increases efficiency in the binding of Ad5-based RPANs to cells expressing αvβ integrins [14]. A single chain T-cell receptor (TCR ) directed against the melanoma-associated antigen in complex with HLA I (major histocompatibility complex) introduced to the C-terminus of pIX also enables RPANs to effectively transduce human melanoma cells [15].

Existing approaches to pIX modification imply a costly and time-consuming generation of RPANs for each targeted receptor. Hence, the development of a universal targeted gene delivery platform based on specific binding of certain molecules to the adenovirus capsid surface, which provides effective targeting of RPANs, is of great interest.

To build this platform, the synthetic domain EE _12_RR _345_L (ER domain) was introduced into the Cterminus of pIX. The ER domain is capable of high efficiency heterodimerization with the partner domain RR _12_EE _345_L (RE domain), yielding a stable structure (leucine zipper). Both synthetic leucine zipper domains were genetically engineered and derived from the appropriate domain of a vitellogenin gene-binding protein (VBP) [16, 17]. Neither of the two 43-amino acid domains forms homodimers even at low temperatures (6 °C and above). However, they heterodimerize under physiological conditions, forming a stable structure “leucine zipper” EE _12_RR _345_L/RR_12_ EE_345_L (or ER /RE ) with the melting point at 73 °C and a dissociation constant* K*_d_ = 1.3 × 10^-11^ M [17].

It should be noted that the approach to the modifying of Ad-based RPANs has already been described [18]. A similar one is used in our work but essentially differs in terms of the choice of the modifiable capsid protein, antibody format, and strategy for vector generation.

We propose integrating the ER domain into pIX as the number of pIX monomers is six times higher than the number of fiber monomers in the Ad5-capsid. Accordingly, more antibodies bind to RPAN in this case, providing more effective penetration of the pIX-modified RPANs into the target cells.

We used single-domain antibodies (nanoantibodies) directed against the carcinoembryonic antigen (CE A) as molecules binding to the modified RPANs and providing targeted gene delivery to specific cells. This choice was determined by a number of the advantages of nanoantibodies; in particular, by the simplicity of genetic manipulations, reduced immune response, favorable pharmacokinetics, good solubility, pH tolerance, and high thermal stability. The nanoantibodies directed against CE A (aCE A-RE ) were selected as this receptor, because they are often found in cancer cells. Furthermore, our experience in the generation of nanoantibodies and their applications, including thee homotrimer of “isoleucine zipper” [19, 20], as well as in the utilization of RPANs for nanoantibody expression *in vivo *[21, 22] significantly contributed to the choice of nanoantibodies.

The aCE A-RE that were used in our work can effectively recognize the CE A–cell surface antigen expressed in the tumor cell lines A549 and Lim1215 to a high level. We have shown that pIX-modified RPANs (Ad5-EGFP-pIX-ER ) carrying aCE A-RE on their surfaces three times more effectively penetrate into the tumor cells lines A549 and Lim1215 via the CAR-independent pathway than unmodified RPANs (Ad5-EGFP) and Ad5-EGFP-pIX-ER (pIX-modified RPANs, which do not have aCE A-RE on their surfaces). We have created the Ad5-EGFP-pIX-ER vector system: a versatile platform for targeted gene delivery, which enables the targeting of particular (tumor) cells by specific binding nanoantibodies directed against a (tumor-specific) surface antigen on the RPAN surface.

## EXPERIMENTAL

**Plasmid vectors**

We used the pBluescript II SK (+) plasmid vector (Fermentas MBI, Lithuania); pGEM-T-Easy plasmid system (Promega, USA); pShuttle-CMV-EGFP shuttle vector containing the cytomegalovirus (CMV) promoter, enhanced green fluorescent protein (EGFP) reporter gene and Ad5 genomic fragments; and pAdEasy-1 plasmid (Stratagene, USA).

**RPAN and bacterial strains**

Ad5-EGFP RPANs comprising the full-length Ad5 genome, and the green fluorescent protein expression cassette under the CMV promoter were generated at the N.F. Gamaleya Research Institute of Epidemiology and Microbiology [23].

The *Escherichia coli *strains DH5α and BJ5183 were used.

**Cell lines**

The following cell lines were used: HEK293 (human embryonic kidney cells containing the Ad5 E1 region), A549 (human lung adenocarcinoma epithelial cell line), H1299 (human non-small cell lung carcinoma cell line), H460 (human lung cancer cell line), H292 (human lung mucoepidermoid carcinoma cell line), Lim1215 (human colon carcinoma cell line), SW480 (human colon adenocarcinoma cell line), and HCT -116 (human colon cancer cell line). The cells were cultured in DMEM (Dulbecco’s modified Eagle’s medium) supplemented with 10% Hy- Clone fetal bovine serum (USA), glutamine, penicillin and streptomycin.

**Enzymes**

Specific restriction endonucleases, T4 DNA ligase, and other enzymes were purchased from Promega (USA), New England BioLabs (USA), Fermentas MBI (Lithuania).

**Cloning of the ER- and RE- leucine zipper domains**

**Table:** PCR primers used for amplification of fragments to clone ER- and RE- leucine zipper domains

Primer	Primer sequence
ER 1F	5'-ccagaactcgagatcgaggcagctttcctggaacgggagaacactgcactgg-3'
ER2F	5'-ccagcgtctgcggaaccgagtctcacagtatcgaactcgttacggacctctg-3'
ER1R	5'-tccgcagacgctggactcgctgccgcagttcagctacacgagtctccagtgcagtgttc-3'
RE1F	5'-ccagaactcgagatccgtgcagctttcctgcgtcaacggaacactgcactgc-3'
RE2F	5'-ccagcgtctggagaacgaagtctcacagtatgaaactcgttacggacctctg-3'
RE1R	5'-tctccagacgctggacctcctgctccagttcagctacctcagtacgcagtgcagtgttc-3'
NotI-Cend-Zipper-rev	5'-cgtacgggtagcggccgctcagaggtccgtaacgag-3'

**Fig. 1:**
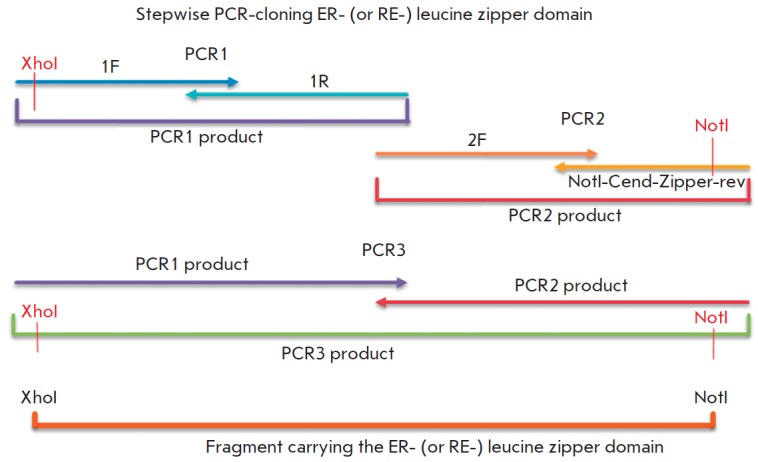
Scheme of the consecutive cloning stages (PCR1, PCR2, PCR3). For convenience of cloning (the introduction of a XhoI restriction site to the 5’-end of the sequence), the third nucleotide residue of the original sequence, G, was replaced by C; the amino acid sequence remained intact

Fragments encoding the ER - and RE - heterodimeric leucine zipper domains were obtained by PCR [17, 18] using the primers listed in *Table* (*Fig. 1*). The amplification with the ER 1F and ER 1R or RE 1F and RE 1R primers (for ER - or RE domains, respectively) resulted in PCR 1 product of 97 bps (PCR 1). A PCR 2 product of 71 bps (PCR 2) was obtained by amplification with primers ER 2F and NotI-Cend-Zipper-rev (for ER domain) or RE 2F and NotI-Cend-Zipper-rev (for RE domain). In the next PCR reaction, we used PCR 1 and PCR 2 products as primers and obtained a PCR 3 product of 154 bps comprising the ER - or RE -domain sequence. The PCR 3 products were then inserted into the XhoI - NotI site of the pBluescript II SK (+) plasmid vector, which resulted in pER and pRE plasmids containing nucleotide sequences encoding the full-length heteromeric leucine zipper domains. The correct insertions of the ER - and RE -domain genes were confirmed by sequencing. For convenience of cloning (to introduce the XhoI site at the 5’-end of the DNA sequence), the third nucleotide of the original sequence, G, was substituted for C. The amino acid sequence remained unchanged.

**Construction of a plasmid carrying the Ad5 genome with deletion of the E1 region,** *EGFP* **cassette, and the sequence of the heteromeric domains of leucine zipper at the C terminus of pIX**

To integrate the ER domain into the C-terminus of pIX, a sequence containing the pIX gene with a deleted stop codon; a spacer (a sequence of the longest α-helix of human apolipoprotein E4 (33 a.a.)) [18], a polylinker carrying the restriction sites BamHI, Kpn2I, NotI, HindIII, AscI and SwaI to insert the target ligands, and the *pIVa2 *gene (from 1 to 832 bps) were synthesized (ZAO “Evrogen”). The synthetic sequence was cloned into the pBluescript II SK plasmid vector to generate plasmid pBssk-pIX-mod containing the *pIX *gene with sites for modifications.

The nucleotide sequence encoding the ER domain was amplified using the BamHI-zipp-forw (5’-ggatcc- ctc-gag-atc-gag-gca-gct-ttc-c-3’) and SwaI-zipprev (5’-att-taa-att-tac-aga-ggt-ccg-taa-cga-gtt-cg-3’) primers, which flanked the 5’- and 3’-regions of the leucine zipper and contained the BamHI and SwaI restriction sites, respectively. The aforementioned plasmid, pER , was used as a template.

The PCR product of 146 bps was cloned into the pGEM-T-Easy plasmid vector. The pGEM-T-ER plasmid was digested with the restriction enzymes BamHI and SwaI, and the sequence encoding the leucine zipper was cloned into the pBssk-pIX-mod plasmid using the same restriction sites. The ApaI-HpaI adenovirus genome fragment containing the modified gene* pIX *was then excised from the pBssk-pIX-ER plasmid and cloned into the pShCMV-EGFP vector at the same sites. Thus, we obtained a pShCMV-EGFP-pIX-ER shuttle vector comprising the sequence encoding the modified pIX with the leucine zipper at the C-terminus, and the *EGFP *reporter gene cassette. This plasmid was linearized by restriction digestion with PmeI and cotransformed together with the pAdEasy-1 plasmid into* E. coli *BJ5183 cells as described in the AdEasy adenoviral vector system (Stratagene, USA). The pAd5-EGFPpIX- ER plasmid was obtained as a result of homologous recombination. It contained the full-length Ad5 genome with deletion of the E1 region, the expression cassette with the *EGFP *reporter gene, and the fragment encoding the leucine zipper at the C-terminus of pIX.

**Production, accumulation, and purification of the pIX-modified RPANs**

The RPANs were produced via transfection of a HEK293 cell line with the pAd5-EGFP-pIX-ER plasmid, which was previously linearized at the PacI restriction site. The transfection was performed with a Metafectene Pro agent (Biontex, Germany). Ad5- EGFP-pIX-ER was accumulated in the HEK293 cell culture. The RPANs were purified and concentrated by cesium chloride density gradient ultracentrifugation of the infected cells lysates. The concentration of the purified RPAN was determined spectrophotometrically (λ = 260 nm) using the conversion factor: 1 OD = 1.12 × 10^12^ viral particles/ml. Ad5-EGFP-pIXER titer was determined using plaque formation assay in the HEK293 cells culture.

**Antibodies**

We used commercial anti-CAR polyclonal antibodies (R&D systems, USA, cat. # AF3336), murine sera containing anti-Ad antibodies obtained after immunization of mice with RPANs, equine secondary antibodies (GE Healthcare, UK), and monoclonal anti-HA antibodies (CHGT-45P-Z, ICL, Inc., USA).

**Generation of aCEA with an additional terminal RE domain**

Generation of single domain mini-antibodies (nanoantibodies) recognizing the carcinoembryonic antigen (aCE A) was performed as previously described [24-28].

**Fig. 2:**
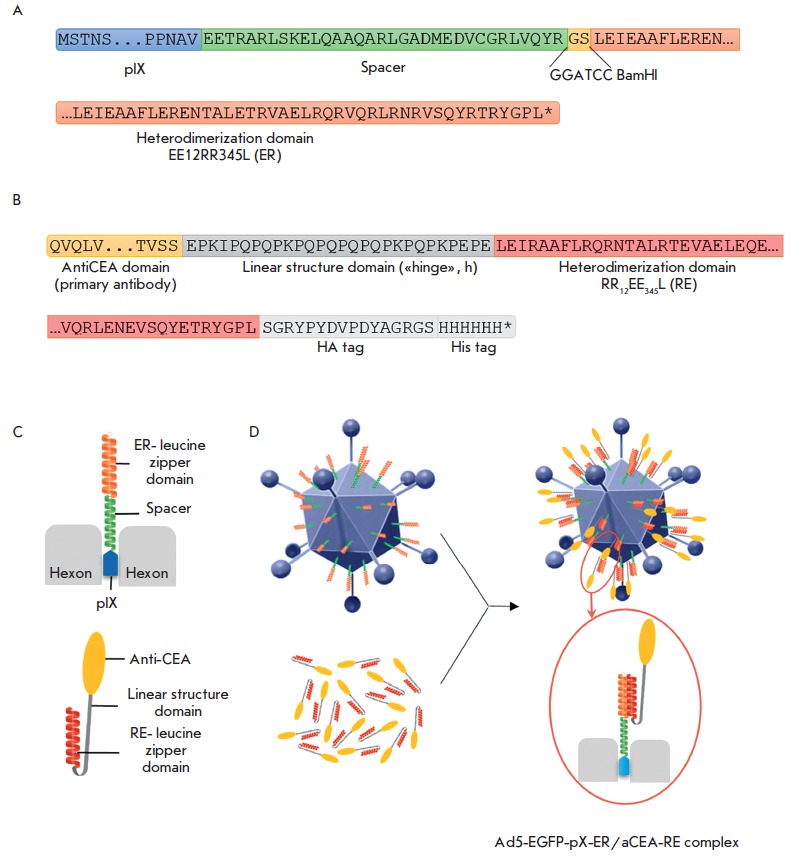
Schematic representation of the recombinant nanoantibody, the structure of the pIX protein modified by the integration of a spacer and the ER- leucine zipper domain, and the formation of a Ad5-EGFP-pIX-ER/aCEA-RE complex with altered tropism. Schemes of the amino acid sequences of the domains of the recombinant pIX (A) and the chimeric nanoantibody aCEA-RE (B). C – positions of the complementary leucine zipper domains; one domain is integrated into the C-terminus of the pIX of Ad5 and protrudes above the capsid surface due to the spacer; another domain is “attached” to the N-terminus of aCEA. D – Formation of the Ad5-EGFP-pIX-ER/aCEA-RE complex through heterodimerization of ER- and RE- leucine zipper domains

**Fig. 3:**
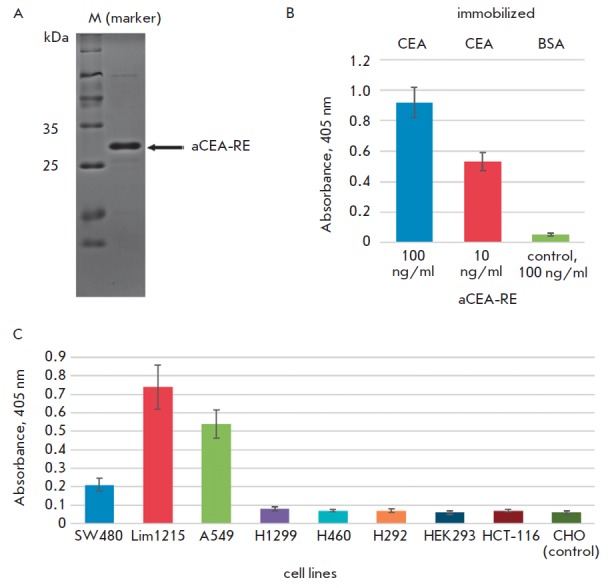
ELISA to detect binding of aCEA-RE to the CEA protein. A – SDS-PAGE of purified aCEA-RE in a 14% SDS-polyacrylamide gel. B – ELISA for detection of aCEA-RE binding to immobilized CEA. The concentrations of aCEARE in assay were 100 ng/ ml and 10 ng/ml. Wells with immobilized bovine serum albumin were used as a negative control. C – ELISA for detection of the aCEA-RE binding to CEA exposed on the tumor cell surface

Bactrian camel *Camelus bactrianus *was immunized sequentially (five times) by subcutaneous injection of an antigen mixed with an equal volume of a complete (for the first injection) or incomplete (for the following injections) Freund’s adjuvant. The antigen, human CE A, was purchased from Xema Medica, Russia (catalog number R224). 0.26 mg of human CE A was used for each injection. The second injection (immunization) was performed three weeks after the initial one, and the following three immunizations were performed every two weeks. Blood (150 ml) was collected five days after the last injection. Then, we isolated RN A from B lymphocytes, synthesized cDNA, carried out two-step PCR and cloning of the amplified sequences encoding nanoantibodies into a pHEN 4 phagemid vector. Selection of cDNA clones encoding nanoantibodies was performed through phage display [24-28]. In this procedure, we used M13KO7 helper phage (New England Biolabs, USA) and human CE A as an antigen immobilized on the bottom of the wells of a 96-well ELISA plate. The same human CE A was used for injections. cDNA from the selected clones was then re-cloned into a new expression vector. Before re-cloning, we added sequences encoding HA and (His)_6_ tags at the 3’-end of the cDNA to increase the efficiency of nanoantibody detection and purification after expression. The specificity and relative affinity of the initially selected nanoantibodies were determined by ELISA via their binding to the immobilized human CE A protein, and, subsequently, to fixed cells overexpressing CE A on the cell surface. Based on the conducted assays, we chose the most effective nanoantibody, anti-CE A/aCE A1. (The antibody sequence, details of its production and analysis are described in the recent patent application # 2,012,113,421, Russian Federation: Tillib, S.V. The single- domain nanoantibody, aCE A1, specifically binding the CE A protein.) To ensure stable binding of aCE A1 to the modified pIX Ad5 (pIX-ER ), the former was modified as described in [20] but a different ligand was used. A RE domain capable of effective dimerization with the ER domain to form a leucine zipper was integrated into aCE A1 instead of the homotrimeric domain (ILZ). Constituents of the modified aCE A1, aCEA-RE , with amino acid sequences, partial for some of them, are shown in *Fig. 2B*. The aCEA-RE accumulating in bacterial periplasm was purified as described previously [20] and detected as an individual band after separation by SDSPAGE in a 14% gel (Fig. 3A).

**ELISA for detection of leucine zipper interactions of recombinant nanoantibodies and pIX RPANs**

A 96-well plate was coated with 2 µg/well of aCEA-RE in a 40 mM potassium carbonate buffer (pH 9.6) at +4° C for 12 h. The plate was then washed three times with 0.05% Tween-20 and three times with distilled water. After Ad5-EGFP-pIX-ER was added at a concentration of 1 µg/ml in the working solution, the plate was incubated in a shaker for 1 h at +37 °C. Ad-EGFP was used as a control. Different dilutions of murine sera in the working solution (1: 800 to 1: 204 800) containing anti-Ad-antibodies were added to the plate and incubated in a shaker at +37° C for 1 h. Following plate washing, horseradish peroxidase (HRP) conjugated anti-species antibodies of working dilution (1: 10000) in phosphate buffered saline (PBS), pH 7.4, with 0.05% Tween-20 were added. TMB substrate was used to visualize the HRP enzymatic reaction; 4 M H_2_SO_4_, to stop it. The optical density of the colored product of the HRP reaction was measured on an iEMS Reader MF (Termo labsystems) at 450 nm.

**Immunohistochemical assay to detect binding of aCEA-RE nanoantibodies to CEA expressed on the surface of tumor cells and to the purified CEA protein**

The ability of aCEA-RE to bind to the human CE A protein immobilized on the surface of microplate wells was examined by the standard ELISA protocol. Microplate wells with immobilized bovine serum albumin (BSA) were used as a control. Anti-HA monoclonal antibodies conjugated to horseradish peroxidase, which were directed against HA-tag at the C-terminus of the aCEA-RE antibodies, were used as secondary antibodies. The activity of horseradish peroxidase was determined using the ABTS chromogenic substrate (2,2’-azinobis(3-ethylbenzothiazoline-6-sulfonic acid)). The optical density was measured at 405 nm using a microplate fluorometer. Control wells (with immobilized BSA) contained no antigen and were blocked and processed together with the experimental wells (with antigen).

The possibility of using aCEA-RE antibody to detect the CE A protein expressed on the tumor cell surface was examined by ELISA on immobilized/fixed cells. The following cell lines were used: A549, H1299, H460, H292, Lim1215, SW480, and HCT -116. The HEK293 cell line (derived from human embryonic kidney cells) served as a negative control as the CE A protein is not detected in this cell line according to published reports. Chinese hamster ovary (CHO) cells were another negative control. The cells were seeded into a 96-well plate at a density of 10^4^ cells per well. A day after seeding, the cells were washed with PBS three times and fixed in 3.7 % formaldehyde diluted in a buffer for 10 min. Fixation was stopped by adding a glycine solution to a concentration of 125 mM. The fixed cells were washed with PBS three times and covered with a blocking buffer, 1% BSA in 1×PBS, for 2 h. The cells were rinsed with 1×PBS and covered with a solution (1×PBS, 0.1 % BSA) containing aCE A-RE nanoantibodies at a concentration of 100 ng/ml. Anti-HA monoclonal antibodies conjugated to horseradish peroxidase were used as secondary antibodies directed against the C-terminal HAtag of the tested aCE A-RE nanoantibody. Horseradish peroxidase activity was determined using a ABTS chromogenic substrate. The optical density was measured at 405 nm using a microplate fluorometer. Control wells (with immobilized HEK293 and CHO cells) were blocked and processed together with the experimental wells.

**Thermal stability assay for pIX-modified RPANs**

HEK293 cells were seeded into 24-well plates at a density of 10^5^ cells per well. After 24 h, the monolayer HEK293 culture was infected with pIX-modified RPAN (10^3^ viral particles per cell in 200 µl of the medium). Before infection, the pIX-modified RPAN were incubated at +37° C and +42° C for 5, 15 and 30 min. The number of fluorescent cells was determined by flow cytometry (Backman Coulter Cytomix FC-500, USA) 24 h after infection.

**Transduction of eukaryotic cells with blocked CAR-receptors by RPANs**

A549 and Lim1215 cells were seeded into a 48-well plate at a density of 2 × 10^4^ cells per well, covered with 10 mg/ml of anti-CAR antibodies and incubated at +37° C for 30 min. The antibodies were removed, the cells were washed and transduced by RPANs (500 viral particles per cell). The RPANs were first pre-incubated with aCE A-RE (240 antibodies per viral particle) for 30 min at +4° C under constant stirring; unbound RPANs were removed. The relative number of fluorescent cells was determined by flow cytometry (Backman Coulter Cytomix FC-500, USA) 24 h after transduction.

## RESULTS AND DISCUSSION

**Construction of recombinant pseudoadenoviral vectors with the modified IX protein**

In 2009, J.N. Glasgow *et al*. conducted a study. They “attached” a leucine zipper domain to the C-terminus of Ad5-fiber to enable specific binding of A5-capsid to single- chain antibodies carrying a complementary leucine zipper domain. Thus, RPANs changed their tropism, providing targeted gene delivery [18]. Our aim was to construct a Ad5-based RPAN bearing a leucine zipper domain at the C-terminus of pIX. We hypothesized that this modification of pIX would provide a more efficient delivery of target genes. Ad5-capsid comprises 240 pIX and 36 fiber monomers. Hence, substantially more anti bodies would bind pIX-modified RPANs than fibermodified RPANs. However, peptide integration into the capsid may cause conformational changes leading to disturbance of RPAN assembly as the C-terminus of pIX is situated between capsid hexons [29]. Therefore, we introduced a spacer of the longest human apolipoprotein E4 α-helix sequence between the C-terminus of pIX and the leucine zipper domain. Such a spacer is the most effective one; it does not significantly affect Ad-assembly as it places the leucine zipper domain above the capsid surface, thus improving the efficiency of Ad5-based RPAN binding to recombinant nanoantibodies, which was demonstrated by J. Vellinga *et al*. [30]. The constructed Ad5-based RPAN with the pIXmodification is schematically shown in *Fig. 2*.

The recombinant vector Ad5-EGFP-pIX-ER encoding the modified pIX protein with a spacer sequence and ER domain of the leucine zipper at the C-terminus was obtained by homologous recombination in *E. coli*.

**Characterization of the Ad5-EGFP-pIX-ER RPAN**

The Ad5-EGFP-pIX-ER RPAN was characterized by the following parameters: concentrations of viral particles and plaque-forming units, thermostability.

The concentration of Ad5-EGFP-pIX-ER was 6.5×10^12^ viral particles/ml, 4.0×10^10^ pfu/ml, while the concentration of the control vector, Ad5-EGFP, was 6.3×10^12^ viral particles/ml, 6.0×10^10^ pfu/ml. These results suggest that the modification introduced into the adenovirus capsid did not affect the efficiency of virion assembly and the vector quality, which is defined by the ratio of viral particles to plaque-forming units (162.5 and 105 for Ad5-EGFP-pIX-ER and Ad5-EGFP, respectively).

One of the problems associated with Ad-capsid protein modifications is destabilization of RPANs. The primary function of a pIX protein is to stabilize interactions between adjacent hexons [31]. Accordingly, modifications of pIX proteins destabilize the capsid structure [32]. Therefore, we examined the structural integrity of virions by comparing the thermal stability of Ad5-EGFP-pIX-ER , the modified vector, and Ad5- EGFP, the unmodified vector, to see whether the ER - leucine zipper domain integrated into the pIX protein affects the structural integrity of the virion.

**Fig. 4:**
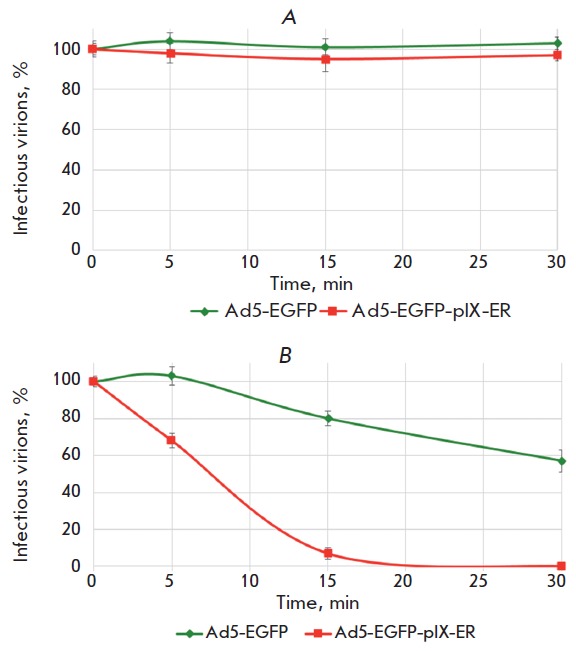
Thermal stability of Ad5-EGFP-pIX-ER. Ad5-EGFPpIX- ER and Ad5-EGFP were incubated at +37°C (A) and +42°C (B) for 5, 15, and 30 min. HEK-293 cells were then infected with 103 viral particles per cell. The number of fluorescent cells was determined by flow cytometry 24 h post infection.

The Ad5-EGFP-pIX-ER and Ad5-EGFP RPANs were incubated at 37 and 42° C for 5, 15 and 30 min; the number of infected cells was determined using a thermal stability assay (*Fig. 4*).

The Ad5-EGFP-pIX-ER and Ad5-EGFP RPANs were incubated at 37 and 42° C for 5, 15 and 30 min; the number of infected cells was determined using a thermal stability assay (Fig. 4).

The Ad5-EGFP-pIX-ER and Ad5-EGFP infectivities did not change when the RPANs were heated up to +37° C for 5, 15, and 30 min. Upon heating at +42° C for more than 5 min, the transduction efficiency for Ad5-EGFP-pIX-ER reduced by 32% and approached 0% if the heating time exceeded 15 min; whereas the Ad5-EGFP infectivity remained the same after 5 min and decreased by 20 and 43% after 15 and 30 min, respectively, under identical conditions. Our data show that the integration of a leucine zipper ER domain into the pIX protein structure reduces the thermostability of Ad5-EGFP-pIX-ER , compared with that of RPANs containing wild-type pIX and are consistent with the published data [33, 34].

**The efficiency in binding the leucine zipper ER domain of the pIX-modified RPANs to the complementary leucine zipper RE domain of recombinant nanoantibodies**

The ability of the leucine zipper ER domain, which was introduced into the pIX protein, to bind to the complementary leucine zipper RE domain of recombinant anti-CE A was defined by ELISA.

**Fig. 5:**
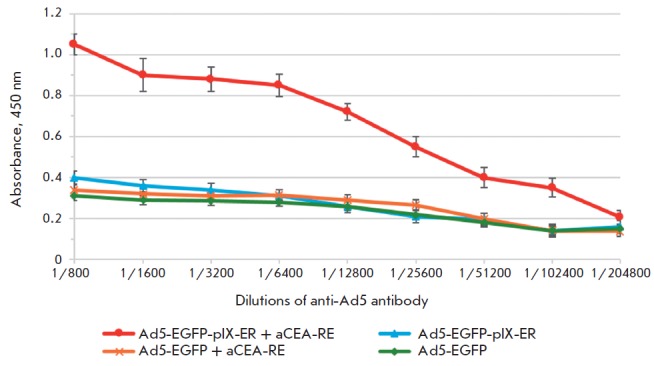
Detection of the Ad5-EGFPpIX-ER binding to aCEA-RE nanoantibodies by ELISA

Wells of a 96-well plate of high adsorption capacity were coated with aCEA-RE . After incubation, unbound antibodies were removed by washing; Ad5-EGFP-pIX ER RPANs were added to the wells. RPANs that did not bind to nanoantibodies were washed away; the formation of an Ad5-EGFP-pIX-ER /aCEA-RE complex was detected using anti-Ad-antibodies (*Fig. 5*).

Thus, we have shown that hydrophobic interactions of the ER - and RE domains of Ad5-EGFP-pIX-ER and nanoantibodies, respectively, to form a leucine zipper provide specific binding of recombinant nanoantibodies to RPANs.

**Selection of cell lines exposing CEA for efficient binding to the aCEA-RE nanoantibody on their surface**

At the next stage of our study, we examined the ability of aCE A-RE nanoantibodies to specifically bind not only to purified CE A, but also to CE A exposed on the cell surface.

*Figure 3B* shows the ELISA results indicating that the aCE A-RE nanoantibody specifically binds to the immobilized human CE A protein at concentrations of 100 and 10 ng/ml. Wells with immobilized bovine serum albumin were used as a control. Signal intensity (optical density at λ = 405 nm) shows the efficiency of nanoantibody binding.

If nanoantibodies recognize the isolated CE A protein, this does not mean that the epitope is accessible for recognition by the nanoantibody when the protein is localized on the cell surface. The possibility to use aCEA- RE nanoantibodies for detecting CE A overexpressed on a tumor cell surface was examined by comparative ELISA for the cell lines SW480, Lim1215, A549, H1299, H460, H292, and HCT -116. The HEK293 and CHO cell lines were used as negative control. The ELISA results are shown in *Fig. 3B*.

Identically to the case of isolated CE A protein, aCE ARE nanoantibody effectively works at a concentration of 100 ng/ml. Specific recognition of A549 and Lim1215 cells occurs due to the high-level expression of CE A residing on the surface of these cells. In contrast, the CE A protein is almost not expressed in control HEK293 cells, which is reflected by the background optical density in the corresponding cells. Similarly, the background signal is observed for the control CHO cells.

As a result, it was shown that the recombinant aCE A-RE nanoantibody is able to specifically interact with two cell lines: A549 and Lim1215. These cell lines were used in further experiments to study the transduction efficiency. At this point, we can solely speculate why only two of the seven tested cell lines specifically interact with the nanoantibody.

This may be caused, for instance, by the different accessibilities of the CE A epitope recognizable by the nanoantibody on the tested cell line surfaces, or by the potential loss of CE A from some cell line surfaces due to uncontrolled prolonged cultivation.

Ad5-EGFP-pIX-ER in complex with aCE A-RE efficiently transduce tumor cells via the CAR-independent pathway

**Fig. 6:**
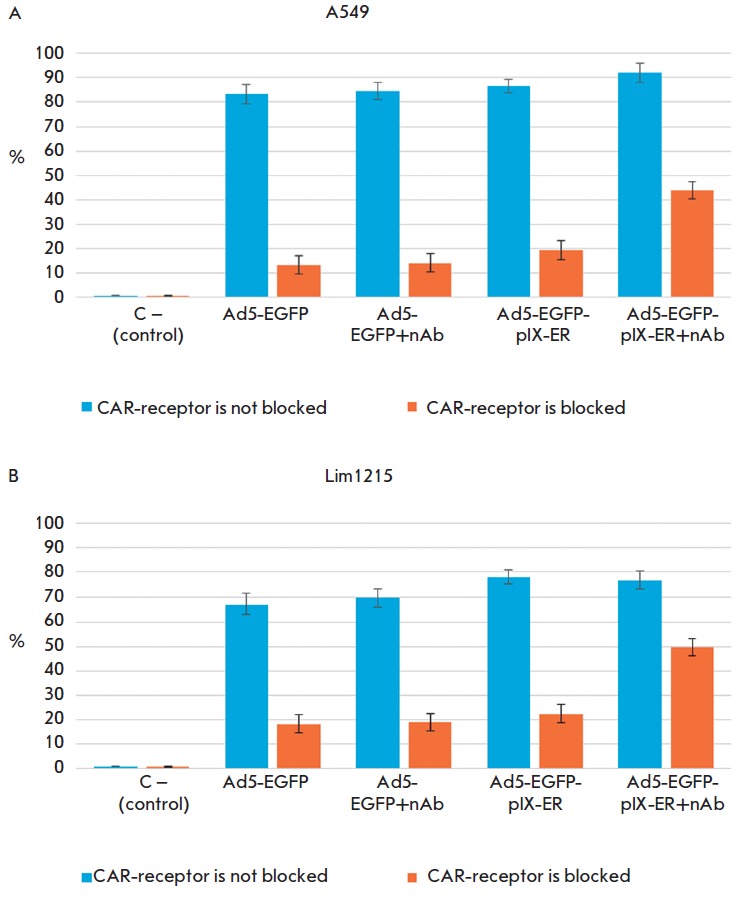
Transduction of tumor cells with Ad5-EGFP-pIX-ER. Cells of the A549 (A) and Lim1215 (B) cell lines were incubated with anti-CAR antibodies at a concentration of 10 mg/ml at +37° C for 30 min. Then, they were infected with Ad5-EGFPpIX-ER and Ad5-EGFP pre-incubated with CEA-RE at a ratio of 1 VP to 240 antibodies at +4°C for 30 min. The used vector dose was 500 VPs per cell. The number of transduced cells was determined by flow cytometry.

At this stage, we examined the effectiveness of penetration of the Ad5-EGFP-pIX-ER /aCE A-RE complex into tumor cells. Due to the fact that the A549 and Lim1215 cell surfaces comprise a large number of CAR receptors [35], native Ad5 receptors, it was necessary to block them. To do so, A549 and Lim1215 cell lines were incubated with 10 mg/ml of anti-CAR-antibodies, and then they were transduced by Ad5-EGFP-pIXER “pre-loaded” with anti-CE A. Ad5-EGFP-pIX-ER (without anti-CE A), Ad5-EGFP and Ad5-EGFP “loaded” with anti-CE A were used as a control (*Fig. 6*).

It was shown that Ad5-EGFP-pIX-ER carrying aCE A-RE on the capsid surface threefold more efficiently transduce A549 and Lim1215 cells than RPAN without bound nanoantibodies under conditions when CAR receptors are blocked. Noteworthy, only 40–60 % of A549 cells in the culture express CE A [36]. Therefore, it can be assumed that the efficiency in the penetration of modified RPANs into tumor cells will be significantly higher when nanoantibodies directed against other tumor-associated receptors or other tumor cell lines are used.

## CONCLUSIONS

We have constructed Ad5-based RPANs with modified pIX proteins carrying leucine zipper domains on the capsid surface. The ability of such Ad5-based RPANs to adsorb nanoantibodies containing complementary leucine zipper domains on their surface has been proved. It has been shown that RPAN with leucine zipper domains “loaded” with aCEA-RE s three times more effectively penetrates into the tumor cells of the A549 and Lim1215 cell lines via the CAR independent pathway than unmodified Ad5-EGFP and Ad5-EGFP-pIX-ER without surface-adsorbed nanoantibodies.

Thus, the results of our work suggest that the vector Ad5-EGFP-pIX-ER can be used as a universal platform that provides targeted gene delivery to particular (tumor) cells by specific binding of nanoantibodies directed against a certain (tumor) surface antigen to the RPAN surface. Any other properly modified protein that specifically recognizes a target of interest can be used instead of nanoantibodies.

## Abbreviations

RPAN: recombinant pseudoadenoviral nanoparticles
CEA: carcinoembryonic antigen
Ad: human adenovirus
Ad5: Ad serotype 5
CAR: coxsackievirus and adenovirus receptor
a.a.: amino acid residue
pfu: plaque-forming unit
